# Changes in Secondary Organic Aerosol Composition and Volatility Going from a Low to a High HO_2_/RO_2_ Regime in α‑Pinene Photooxidation

**DOI:** 10.1021/acsestair.5c00254

**Published:** 2025-12-03

**Authors:** Veronica Geretti, Yarê Baker, Thomas Bannan, Aristeidis Voliotis, Quanfu He, Thorsten Hohaus, Sungah Kang, Michael Priestley, Epameinondas Tsiligiannis, Hui Wang, Rongrong Wu, Annika Zanders, Sören R. Zorn, Gordon McFiggans, Cheng Wu, Thomas F. Mentel, Mattias Hallquist

**Affiliations:** 1 Department of Chemistry and Molecular Biology, 3570 University of Gothenburg, Gothenburg 413 90, Sweden; 2 Institute for Climate and Energy Systems, ICE-3 Troposphere, Forschungszentrum Juelich GmbH, Juelich 52428, Germany; 3 Department of Earth and Environmental Sciences, 5292 University of Manchester, Oxford Road, Manchester M13 9PL, U.K.

**Keywords:** secondary organic aerosols, photooxidation, α-pinene, composition, volatility, hydroperoxyl radical, peroxyl radical

## Abstract

The mechanisms of secondary organic aerosol (SOA) formation are not yet fully understood. The relative abundance of hydroperoxyl radicals (HO_2_) and peroxy radicals (RO_2_) affects SOA properties, but chamber experiments often underemphasize the role of HO_2_. To clarify their contribution, this study compares the composition and volatility of SOA formed by the hydroxyl radical (OH) oxidation of α-pinene under low and high HO_2_/RO_2_ regimes with a constant OH concentration. The particle-phase was characterized with a Filter Inlet for Gases and AEROsols coupled to an iodide Chemical Ionization Mass Spectrometer (FIGAERO CIMS), and a CIMS with NO_3_^–^ ionization was used for gas-phase measurements. High HO_2_/RO_2_ conditions weakened the particle-phase monomer (C_10_), fragment (C_4–9_), and accretion product (C_11–20_) signals by 34%, 29%, and 78%, respectively, compared to low HO_2_/RO_2_ conditions. The only species with an increased signal (180%) was C_10_H_18_O_7_. The gas-phase changes align with those in the particle-phase within a factor of 2. Overall, the organic mass was reduced by 47% and 39% for particle and gas-phases, respectively. Bulk SOA volatility (log C*) increased slightly from −0.22 μg m^–3^ to −0.1 μg m^–3^, reflecting the suppression of low volatility accretion products but formation of high volatility hydroperoxide monomers. This study highlights the importance of HO_2_ for SOA formation and model predictions.

## Graphical abstract

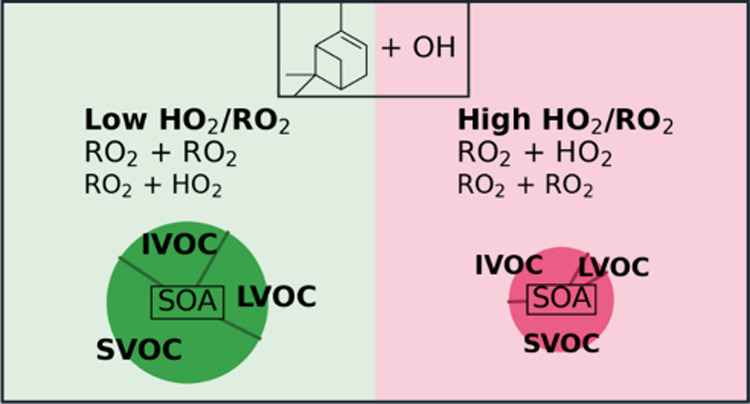

## 1 Introduction

Secondary organic aerosol (SOA) is a significant climate forcer, impacting Earth’s radiative balance through both direct and indirect effects.^1^ The current understanding of SOA formation is insufficient for fully quantitative and predictive modeling in atmospheric systems.^2–4^ This knowledge gap presents a significant research challenge in atmospheric science and climate research. SOA is formed by the oxidation of volatile organic compounds (VOCs) in the atmosphere.^5^ Globally, the biogenic emissions of VOCs far exceed the anthropogenic ones.^6^ The main biogenic SOA precursors are isoprene (C_5_H_8_) and monoterpenes (C_10_H_16_), which account for around half and 15% of total VOC emissions, respectively.^7^ The most abundant monoterpene is α-pinene, which comprises almost 45% of the world’s estimated annual monoterpene emissions.^8^ Atmospheric oxidation of α-pinene forms peroxyl radicals (RO_2_) that can react with other R’O_2_ species or hydroperoxyl radicals (HO_2_) in the absence of NOx, leading to the formation of carbonyls (R=O), alcohols (R–OH), accretion products (ROOR’), and alkoxy radicals (R’O) via Reactions 1–3, as well as hydroperoxides (ROOH) via Reaction 4.

$${\mathrm{RO}}_{2}+R^{’}O_{2}\to R-\mathrm{OH}+R=O+O_{2}$$ R1

$$\mathrm{RO}+R^{’}O_{2}\to \mathrm{RO}+R^{’}O+O_{2}$$ R2

$${\mathrm{RO}}_{2}+R^{’}O_{2}\to {\mathrm{ROOR}}^{’}+O_{2}$$ R3

$${\mathrm{RO}}_{2}+{\mathrm{HO}}_{2}\to \mathrm{ROOH}+O_{2}$$ R4

Previous studies have shown that α-pinene–OH oxidation mainly produces C_10_H_17_O_*x*_ and C_10_H_15_O_*x*_ peroxyl radicals. First-generation C_10_H_17_O_*x*_ radicals form after the addition of OH to the α-pinene double bond, followed by O_2_ attack (MCM v.3.3.1).^9,10^ C_10_H_15_O_*x*_ radicals can form via α-pinene ozonolysis, in which case they are produced by decomposition of Criegee intermediates to yield C_10_H_15_O_4_.^11^ Alternatively, they can form via H abstraction resulting from a second OH attack on the first-generation product pinonaldehyde, C_10_H_16_O_2_.^9,10,12^ C_10_H_15_O_*z*_-RO_2_ can react with the same or another RO_2_ species (cross-reactions 1–3) to form closed-shell products (C_10_H_14_O_*z*–1_ carbonyls or C_10_H_16_O_*z*–1_ alcohols) or with HO_2_ to form C_10_H_16_O_*z*_ hydroperoxides. Conversely, C_10_H_17_O_*z*_-RO_2_ species undergo cross-reactions to produce C_10_H_16_O_*z*–1_ carbonyls and C_10_H_18_O_*z*_ alcohols, or react with HO_2_ to form C_10_H_18_O_*z*–1_ hydroperoxides.^13,14^ Some peroxy radicals undergo autoxidation, leading to highly oxidized molecules (HOM, ≥6 oxygen atoms) formation (see Bianchi and references within).^15^ HOM-RO_2_ undergo bimolecular reactions when a lack of abstractable hydrogens makes autoxidation uncompetitive.^16^ Peroxy radical cross-reactions can generate alkoxy radicals (RO) that react via three primary pathways: reaction with O_2_ (a minor channel), isomerization, and fragmentation.^17^ In the isomerization pathway, alkoxy radicals undergo rapid hydrogen shifts to form new peroxy radicals, thereby altering their oxygen parity compared to their parent RO_2_ species.^14,18^ The fragmentation pathway produces molecules with lower carbon numbers than the parent RO_2_.^19^ The formation of accretion products is not yet fully understood but proceeds via mechanisms involving recombination reactions of “conventional” RO_2_, two HOM-RO_2_, or a combination of both.^20–23^

Docherty and Ziemann^24^ and Keywood et al.^25^ showed that the HO_2_/RO_2_ ratio can strongly affect the volatility distribution of VOC oxidation products and thus influences SOA yields. In the atmosphere this ratio may be below or above one, but values above one are common in both urban and rural environments.^26–32^ SOA production from VOCs has been studied extensively using chamber experiments.^33–38^ However, Schervish and Donahue^39^ noted that such experimental systems typically have negligible levels of HO_2_ and may thus overestimate SOA formation potential because a predominance of large RO_2_ radicals would favor R3-type dimerization reactions that produce high molecular weight products and enhance SOA formation.^20^ By comparing ambient and chamber data, Kourtchev et al.^40^ showed that elevated BVOC levels enhance ROOR’ formation, linking high precursor concentrations to increased dimer/trimer production. Chen et al.^41^ observed environmentally significant SOA distribution at the lowest VOC concentration investigated. Realistic levels of HO_2_ radicals (which favor [Disp-formula eq4]) are thus essential to accurately reproduce atmospheric SOA formation conditions. This conclusion is supported by calculations reported by Bonn et al.,^42^ indicating that on a global scale, hydroperoxides are the main products of monoterpene oxidation, while computational studies on α-pinene oxidation by OH suggest that hydroperoxide yields for typical atmospheric α-pinene concentrations are 90% in low-NOx systems.^43^ Accordingly, Eddingsaas et al.^44^ found that hydroperoxides were the main first-generation oxidation products of α-pinene photolysis under low NOx conditions, making RO_2_ + HO_2_ the dominant peroxy radical reaction. Moreover, measured atmospheric concentrations of accretion products (ROOR’) in gas- and particle-phases typically indicate that these species are less abundant than in chamber experiments^45–47^ and may also have different chemical compositions due to the more varied precursor pool in the ambient.^48^

One may note that the effect and direction of the HO_2_/RO_2_ ratio on SOA formation may differ depending on the reaction system and experimental conditions.^49^ For example, Henry and Donahue^50^ reported that SOA yields increased with a higher HO_2_/RO_2_ ratio during α-pinene ozonolysis. According to Keywood et al.,^25^ whether an elevated HO_2_/RO_2_ ratio enhances or suppresses SOA production depends on the position of the alkene double bond: endocyclic alkenes generally favor increased SOA formation, whereas exocyclic alkenes tend to reduce it. Since α-pinene contains an endocyclic double bond, higher SOA production would be expected under elevated HO_2_/RO_2_ conditions, consistent with the observations of Henry and Donahue.^50^ Nevertheless, Schervish and Donahue et al.^39^ and Baker et al.^51^ demonstrated the opposite effect, with SOA yields decreasing as the ratio increases. It is thus critical to further investigate under various conditions with different methods the effect of changes in chemical regimes on SOA formation.

To establish more realistic experimental SOA formation conditions, Schervish and Donahue^39^ simulated an elevated HO_2_/RO_2_ regime under dark ozonolysis conditions by adding carbon monoxide (CO) as a proxy for α-pinene to generate HO_2_ radicals. This yielded a product distribution with reduced levels of low and extra low volatility products, such as accretion products, and increased levels of hydroperoxyl functionalities. McFiggans et al.^52^ observed reduced accretion products and SOA yields when α-pinene was mixed with isoprene, CH_4_, or CO. This suppression was attributed to the scavenging effect of isoprene small chained RO_2_, CH_3_O_2_, and HO_2_. However, an OH scavenging effect was observed, which reduced the oxidant availability to α-pinene, thereby limiting RO_2_ radical and product formation. McFiggans et al.^52^ proposed an innovative way of studying these effects by adjusting oxidant levels after a perturbation that returns them to their original values.

Given the sparse and contradictory results on the effect on SOA depending on experimental conditions, there is a need to improve our understanding of how the HO_2_/RO_2_ ratio affects the composition and volatility of SOA to enable more accurate modeling. From our previous findings on the effects on the gas-phase production from a change in the HO_2_/RO_2_ ratio, we now formulate the hypothesis that we should observe corresponding changes also in the particle phase directly influencing SOA volatility. The same design was used with a controlled chamber study on OH-initiated α-pinene oxidation with two different HO_2_/RO_2_ ratios under constant oxidant levels. A low α-pinene concentration (10 ppb) was used to approximate atmospheric levels.^53–57^ The experiments were conducted in the SAPHIR-STAR continuously stirred tank reactor at the Forschungszentrum Juelich. SOA composition was characterized by using a Filter Inlet for Gases and AEROsols (FIGAERO) coupled with a high-resolution time-of-flight chemical ionization mass spectrometer (HR-ToF-CIMS). Gas-phase HOM chemistry was analyzed using a ToF-CIMS coupled to a multischeme ionization inlet (MION), with NO_3_^–^ as the reagent ion. An extensive evaluation and description of this system’s gas-phase HOM chemistry were published by Baker et al.^51^ Particle and gas-phase oxidation products’ levels were compared between a low (1:100) and a high (1:1) HO_2_/RO_2_ regime. Changes in the particle-phase due to the regime change were compared to those in the gas-phase. The volatility of SOA components was compared between the two regimes using a combined approach based on maximum desorption temperature (Tmax) and log C* parametrization.

## 2 Materials and Methods

### 2.1 Chamber Set Up and Experiments

The SAPHIR-STAR is an upgraded version of the JPAC chamber,^58^ with programmable control and real-time monitoring of all parameters. Briefly, the chamber is a borosilicate glass cylinder with a volume of 2000 L and is operated as a continuously stirred tank reactor. The inflow was set 32 LPM, giving an average residence time in the chamber of approximately 1 h. The results presented here pertain exclusively to steady-state conditions. All experiments were performed at a relative humidity of 50% and at 20 °C, ensured by the climate-controlled surroundings. Ozone was generated by a self-built device using a pen ray lamp for generating O_3_ by O_2_ photolysis and was monitored with an O342e ozone analyzer (Envea GmbH). The OH radicals were produced via ozone photolysis in the presence of water vapor in the chamber by using two UV–C lamps with a wavelength of 254 nm. The lamps could be covered with a shield, allowing their intensity to be adjusted by varying the percentage of coverage. The lamps’ variable coverage and slight O_3_ inflow adjustments permitted the control of OH production. More details about the SAPHIR-STAR and its operation are available elsewhere.^51^

α-Pinene (≥99% purity, Sigma-Aldrich Merck KGaA) was injected with a syringe pump (Fusion 4000, CHEMYX Inc.) into a heated glass bulb and then introduced into the main flow to the chamber by a 1 LPM N_2_ flow. The α-pinene concentration was measured via proton transfer reaction mass spectrometry (PTR-QMS; Ionicon GmbH). To increase HO_2_ levels and thus the HO_2_/RO_2_ ratio, CO was added from a gas bottle (10% CO in N_2_, Messer SE & Co. KGaA) to raise its concentration in the chamber to 2.5 ppm before reacting. In this context, CO serves as a proxy for any atmospheric compound whose oxidation produces HO_2_ radicals via OH oxidation: $\mathrm{CO}+\mathrm{OH}\overset{O_{2}}{\to }{\mathrm{HO}}_{2}+{\mathrm{CO}}_{2}$ .

Two distinct chemical regimes were examined: a low HO_2_/RO_2_ regime (typical for chamber experiments) where RO_2_ species predominate (HO_2_/RO_2_ ratio ≈ 1:100) and a high HO_2_/RO_2_ regime established by adding CO, with a HO_2_/RO_2_ ratio of around 1:1. To maintain constant α-pinene OH reactivity (i.e., OH turnover), the OH concentration was adjusted after addition of CO such that the α-pinene reactivity remained the same under both regimes.

To simulate the HO_2_/RO_2_ ratios, box model calculations were performed with the FZJ institute’s software package (EASY Version 5.69b), applying the MCM v3.3.1 chemistry^9,10^ under the conditions of the SAPHIR-STAR chamber. More information is available elsewhere.^51^ Model-based predictions indicated that the addition of CO reduced the abundance of RO_2_ radicals by ≈70% and increased that of HO_2_ radicals by ≈50% in all experiments. However, as these values could not be experimentally confirmed, they should be taken as rough estimates only.

Four steady state experiments were performed for both low and high HO_2_/RO_2_ regimes. Seed particles were used to provide a surface for condensation to enable particle-phase characterization (experiments 1 and 2). To this end, ammonium sulfate (≥99% purity, Merck KGaA) seed particles were produced by a TSI atomizer (model 3076, TSI GmbH) and dried to 50% relative humidity to match the conditions in the chamber. Experiments were also done without seed particles (experiments 3 and 4) to study gas-phase chemistry in the absence of particle mass transfer by condensation. The experimental conditions are summarized in Table 1. A Scanning Mobility Particle Sizer (SMPS; Model 3080, TSI GmbH) with a CPC (Model 3788, TSI GmbH) was used to measure the particle size distribution and number concentration of the produced aerosol. A high-resolution aerosol mass spectrometer (HR-TOF AMS; Aerodyne Inc.)^59^ was used to measure aerosol composition, but a problem with the aerodynamic focusing lens affected the AMS, resulting in a significant shift in the lower cutoff and, consequently, in a significant reduction of around 40% in the measured aerosol mass. Therefore, AMS measurements were excluded from the analysis presented here due to the high uncertainty and the very low measured organic fraction. Still, the AMS data were suggesting similar trends to those reported in Baker et al.^51^

**Table 1:** Experimental Conditions for Low and High HO_2_/RO_2_ Regimes with (1 and 2) and without Seeding (3 and 4)^a^

HO_2_/RO_2_	α-pin in (ppb)	α-pin SS (ppb)	HO_2_ (cm^–3^) ×10^7^	RO_2_ (cm^–3^) ×10^9^	OH (# cm^–3^) ×10^6^	α-pin OH turnover (cm^3^ s^–1^) ×10^7^	pinonaldehyde (# cm^–3^) ×10^9^	(NH_4_)_2_SO_4_ (μg/m^3^)
Exp.1 Low	7.1	2.7	3.6	4.7	4.7	1.7	5.1	11.6
Exp.2 High	8.1	3.4	200	1.2	3.4	1.6	2.2	10.7
Exp.3 Low	8.0	2.6	3.7	5.0	5.7	2.0	6.1	
Exp.4 High	8.5	3.5	200	1.3	3.4	1.6	2.3	

a From left to right: α-pinene concentration (ppb) in the empty chamber, α-pinene concentration (ppb) in the steady state (SS), estimated concentrations of HO_2_ and RO_2_ in the SS, calculated OH calculated concentration in the SS, calculated α-pinene turnover in the SS, modeled pinonaldehyde concentration, and the seeds’ mass concentration from SMPS measurements in the SS, using a density of 1.38 g/cm^3^.^60,61^

The gas- and particle-phases were characterized using two CIMS systems that are described in the following section. Our analysis is based on particle-phase data gathered using the FIGAERO CIMS during seeded experiments 1 and 2 and gas-phase data gathered using the NO_3_^–^ CIMS in unseeded experiments 3 and 4.

### 2.2 Chemical Ionization Mass Spectrometry

A high-resolution time-of-flight chemical ionization mass spectrometer (HR-ToF-CIMS) coupled with a Filter Inlet for Gas and Aerosols (FIGAERO inlet) measured both gas- and particle-phase chemical composition with iodide reagent ions, denoted as FIGAERO CIMS. The FIGAERO CIMS operates in negative ionization mode with iodide (I^–^) as a reagent ion, which forms iodide clusters with the analyte molecules (MI^–^). Iodide ions are generated by ionizing methyl iodide (CH_3_I) using a polonium-210 radioactive source.^62–64^

In this study, the FIGAERO particle-phase inlet was connected to the SAPHIR-STAR chamber via a 40 cm long glass tube with an inner diameter of 1 cm. Particle sampling was performed with an inflow of 4 LPM. The FIGAERO inlet collected particles on a filter for 1 h. In the following desorption process, the filter temperature was increased gradually using a heated 2 LPM flow of ultrahigh purity N_2_. The desorption phase involved 20 min of temperature ramping from room temperature to 200 °C at about 9 °C/min, then 20 min of soaking at 200 °C, and 10 min of cooling. The outflow from the FIGAERO inlet entered the Ion Molecule Reactor (IMR) where the product clustered with the iodide ions. The IMR was humidified during the desorption phase so that the IH_2_O^–^ to I^–^ ratio remained the same as that during sampling from the chamber for consistent sensitivity. Each steady state was maintained for 3 h, enabling the FIGAERO to complete two sampling cycles. The variation between the two sampling cycles was below 5%, and the data presented in this study was the average of two cycles.

Due to the low VOC concentration applied in this study and unavoidable losses of low volatility vapors due to sampling lines, many gas-phase signals detected by the FIGAERO CIMS were close to the detection limit, and 80% of the total signal had a signal-to-noise ratio (SNR) of ≤2. Thus, we present gas-phase measurement utilizing an atmospheric-pressure-interface time-of-flight mass spectrometer (APi-TOF-MS, Tofwerk AG)^65^ with a long TOF (LTOF) coupled to a Multischeme chemical IONization Inlet (MION, Karsa Oy), for a lower detection limit.^66^ For this study, the nitrate (NO_3_^–^) chemical ionization scheme was used. Compared to the I^–^ reagent ion, NO_3_^–^ is more sensitive toward HOM, i.e., lower volatility compounds that are expected to condense with seeds.^67,68^ This combination provides overlapping coverage of the SVOC (semivolatile organic compounds), LVOC (low volatile organic compounds), and ELVOCs (extremely low volatile organic compounds) regions,^69,70^ making it a valuable tool for determining whether changes in the gas-phase are reflected in the particle-phase. The APi-TOF-MS is henceforth referred to as the NO_3_^–^ CIMS. Further details on the operation of the NO_3_^–^ CIMS are available elsewhere.^51^

To compare data from two instruments, the analysis focused on relative rather than absolute signal strengths and determined that all compounds were detected with equal sensitivity within each instrument. For example, differences in product distribution between the low and high HO_2_/RO_2_ regimes were assessed by calculating the ratios of each compounds’ signal strengths under the two regimes (high HO_2_/low HO_2_). This approach partly overcomes problems arising from differences in the sensitivity of the two. However, the two detection techniques may be differently sensitive toward isomers, i.e., the same molecular formula but different structures, so the gas- and particle-phase observations of the compounds with the same molecular formula are not expected to precisely reflect one another.

#### 2.2.1 FIGAERO–CIMS Data Treatment

Raw data with a time resolution of 1 s were analyzed using the Tofware v4.0.2 (www.tofwerk.com/tofware), developed for Igor Pro 8. The masses used to convert time-of-flight to *m*/*z* were I^–^ (126.904 *m*/*z*), IH_2_O^–^ (144.915 *m*/*z*), ICH_2_O_2_^–^ (172.909 *m*/*z*), IC_2_H_4_O_2_^–^ (186.926 *m*/*z*), IC_3_H_6_O_3_^–^ (216.936 *m*/*z*), ISO_3_^–^ (206.861 *m*/*z*), and H_2_SO_4_I^–^ (224.871) (only in seeded experiments), C_10_H_16_O_5_I^–^ (343.004 *m*/*z*), and IC5HF9O2- (PFPeA, 390.888 *m*/*z*). The average spectral resolution was 3500 m/Δm. The molecular formulas of the detected compounds were determined through high-resolution multipeak fitting analysis. It should be noted that each identified molecular formula may correspond to multiple isomers. Data was extracted, normalized against the sum of the I^–^ and IH_2_O^–^ signals and multiplied by 10^6^. For the analysis, the signal was further normalized against the α-pinene turnover (Table 1) as described by Baker et al.^51^

The FIGAERO multipeak thermograms were deconvoluted using an updated version of GUFIT (Gothenburg University FItting for Thermograms^71,72^ ), which is described in detail in Section S2 of the Supplemental Data. Briefly, GUFIT uses a varying number of Exponentially Modified Gaussian functions (EMGs) (here, 1 to 4) to model a thermogram’s shape. The first Gaussian (EMG1) has the lowest Tmax among the EMGs. Its Tmax increases with *m*/*z*, consistent with values reported in the literature for a specific chemical composition^62,73–76^ (Figure S1). EMG peaks at higher Tmax values (EMG2, EMG3, and EMG4) may arise from the presence of isomers,^62,77^ thermal degradation of accretion products,^74,76,78–82^ or from ammonium sulfate’s decomposition^76^ or interactions with organic compounds on the filter.^62,83^ To avoid the influence by these processes, the analysis was based on the relative change in the signal area of EMG1.

Before applying GUFIT, the particle-phase signal was corrected by subtracting the filter background signal (see information about background subtraction in Section S1). After applying GUFIT, the selected EMG peak area(s) corresponding to the assigned product were integrated using the trapezoidal method and weighted for particle-phase collection inflow rates and sampling duration.

#### 2.2.2 Volatility Estimation

To estimate the volatility of SOA components, a combined approach based on Tmax and log C* parametrization from Peräkylä et al.^84^ was used. Peräkylä et al.^84^ parametrization was developed based on experimental data of the condensation of gas-phase organic vapors from an α-pinene system. By adding ammonium sulfate seeds and observing the gas-phase reduction due to condensation, they estimated the individual saturation concentration C*. The saturation concentration is calculated in the following way:

$$\log_{10}(C^{*}[\mu g\;m^{-3}])=0.18\times \mathrm{nC}-0.14\times \mathrm{nH}-0.38\times \mathrm{nO}+0.80\times \mathrm{nN}+3.1$$ 1

where nC, nH, nO, and nN are the numbers of carbons, hydrogens, oxygens, and nitrogen of the molecule, respectively.

The Peräkylä et al.^84^ parametrization was used here, among others,^85–88^ motivated by being developed based on an α-pinene oxidation system with ammonium sulfate seeds. Moreover, the parametrization is most efficient from the SVOC to the LVOC range, which is primarily the range expected from evaporation in the utilized FIGAERO inlet. A detailed discussion on various parametrization and estimation of vapor pressures was recently presented by Ylisirniö et al.^89^

The Tmax is related to the products’ vapor pressure, which depends on their chemical and physical properties.^62,71,75,80^ Consequently, it can provide information about the volatility of isomeric products, which is impossible with methods based on chemical composition alone. However, obtaining absolute C* values from Tmax measurement can be challenging, and one needs a method to calibrate the relationship. In the absence of authentic standards with known vapor pressures (pure α-pinene oxidation products are hard to derive commercially, and they do not cover the full range of volatilities), an alternative way was used. We took advantage of an observed linear relationship between parametrized log C* from Peräkylä et al.^84^ and measured Tmax (see supplement Section S4). From this relationship, C* could be derived for each individual Tmax measured and sorted into volatility bins.

## 3 Results and Discussion

The impact on SOA formation of shifting from a low to a high HO_2_/RO_2_ regime was assessed by observing the resulting changes in the distribution of α-pinene oxidation products. Chemical components detected by FIGAERO CIMS were classified into three major categories: monomers (C_10_H_14,16,18_O_2–10_), fragments (C_4–9_H_4–18_O_2–10_), and accretion products (C_11–20_H_12–34_O_2–10_). We identified 219 peaks in total: 24 monomers, 95 fragments, and 100 accretion products. The particle-phase signal discussed here is based on the area of the thermograms represented by the fitted Exponentially Modified Gaussian at the lowest Tmax (EMG1 area; see Section S2). The impact of a high HO_2_/RO_2_ regime including other EMGs is presented in the supplement Section S3. Generally, the particle-phase signal decreases at high HO_2_/RO_2_, most evidently in the accretion product region, while the trend in the fragment and monomer region is less distinct (Figure 1).

**Figure 1:**
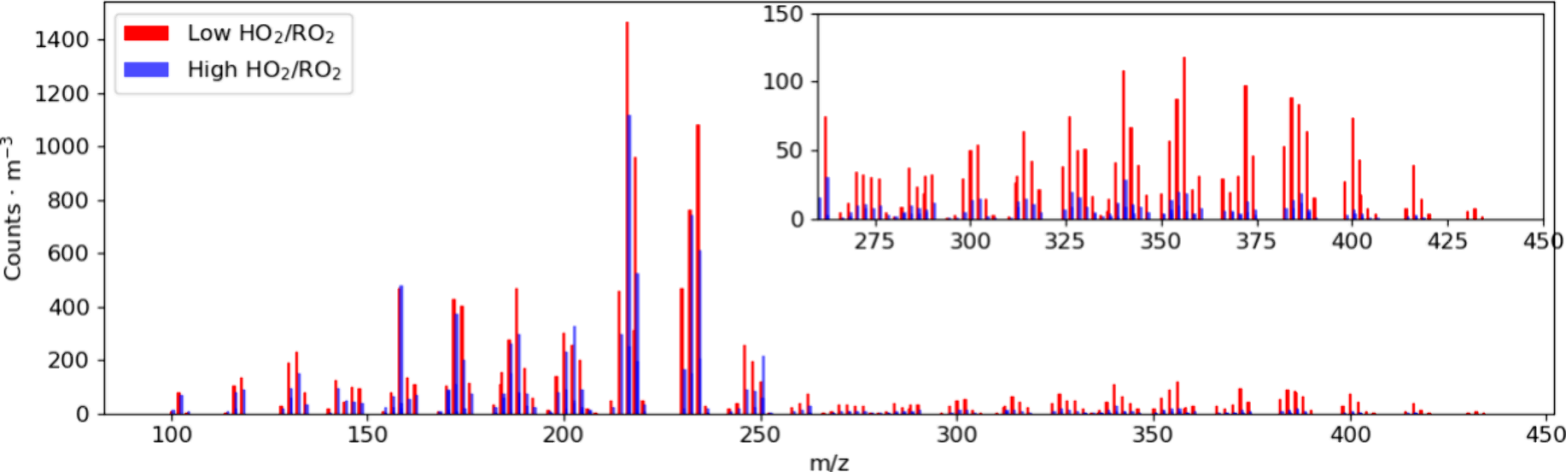
Mass spectra of α-pinene particle-phase signals from the FIGAERO CIMS measurements. The red and blue bars are normalized particle-phase product signals under low and high HO_2_/RO_2_ regimes, respectively. Data from exp. 1 and 2.

Although the α-pinene turnover was held constant in all experiments by adjusting the OH concentration, the RO_2_ sink is enhanced in the high HO_2_/RO_2_ regime because *k*_RO2HO2_ (10^–11^ cm^3^ molecule^–1^ s^–1^) is faster than *k*_RO2RO2_ (10^–12^ cm^3^ molecule^–1^ s^–1^).^90^ On the basis of these general rate coefficients and the radical concentrations from the model of Baker et al.,^51^ the contributions of RO_2_ + R’O_2_ reactions were determined to be ≈96% at low HO_2_/RO_2_ and ≈10% at high HO_2_/RO_2_. The corresponding values for RO_2_ + HO_2_ reactions were ≈4% and ≈90%, respectively.

The fate of “conventional” RO_2_ is to form monomers, alkoxy radicals, or dimers via [Disp-formula eq1], 2, 3, or 4. The RO_2_ cross-reactions are expected to become less significant at high HO_2_/RO_2_ ratios, making RO_2_ + HO_2_ reactions the main chemical sink of RO_2_. However, autoxidation of RO_2_ can be 100 times faster than both *k*_RO2RO2_ and *k*_RO2HO2_,^13,91–93^ so differences in HOM formation between high and low HO_2_/RO_2_ regimes result only from reductions in the RO_2_ concentration.

High HO_2_/RO_2_ conditions generally weaken the particle-phase signal, particularly in the accretion products region; the trend in the fragments and monomers regions is less distinct (Figure 1). Note that the iodide reagent ion’s selectivity may prevent detection of some products formed under high HO_2_ conditions because some products may have low adduct-forming capacity (e.g., due to low polarity). Moreover, some products may have high enough volatility that prevents their partitioning into the particle-phase. Therefore, while high HO_2_ levels seem to generally reduce the oxidation product abundance, this may not be true for products not readily detected under the ionization scheme applied here.

### 3.1 Impact on SOA Product Distribution

Under high HO_2_/RO_2_ conditions, the monomer, fragment, and accretion product signals were reduced by 34%, 29%, and 78%, respectively, relative to the low HO_2_/RO_2_ regime (Figure 2). Suppression of the accretion product signal at high HO_2_/RO_2_ ratios was also observed by Kang et al., McFiggans et al., and Schervish and Donahue.^14,39,52^ The particularly large decrease in accretion products may be because their formation occurs partly via a second-order reaction in the gas-phase ([Disp-formula eq3]), giving rise to a quadratic relationship. Another potential accretion product formation pathway involves initial formation of acyl peroxy radicals by OH oxidation of pinonaldehyde, followed by H-abstraction and O_2_ addition.^23^ High HO_2_/RO_2_ conditions reduce the pinonaldehyde concentration because the comparatively low concentration of alkoxy radicals reduces the abundance of acyl peroxy radicals derived from the OH-oxidation of pinonaldehyde, further reducing accretion product levels.

**Figure 2:**
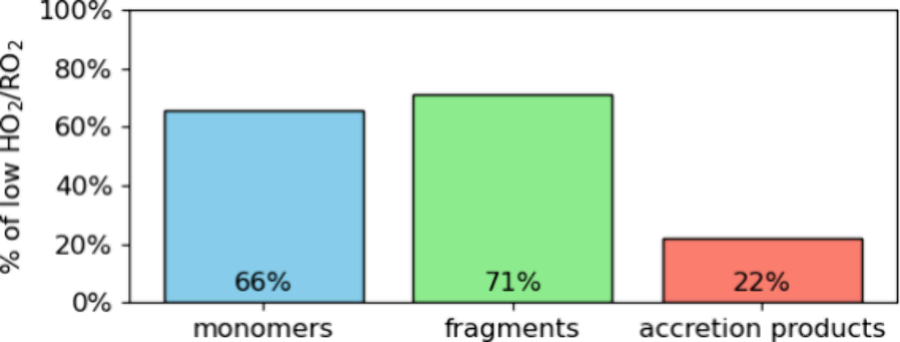
Differences in the particle-phase summed signal of α-pinene products from α-pinene OH oxidation under the high HO_2_/RO_2_ regime relative to the low HO_2_/RO_2_ regime. Products are classified based on their number of carbons: 4 ≤ C ≤ 9 for fragments, C = 10 for monomers, and 17 ≤ C ≤ 20 for accretion products. Data from exp. 1 and 2.

Compared to the low HO_2_/RO_2_ regime, the high HO_2_/RO_2_ regime was expected to reduce the relative abundance of carbonyl and alcohol monomers formed via [Disp-formula eq1] while increasing that of hydroperoxide monomers formed via [Disp-formula eq4].^39,51^ In order to identify these functional group changes, the α-pinene monomers were grouped into three families whose members all have the same CH composition, variable numbers of oxygens, and 14, 16, or 18 hydrogens.

Under high HO_2_/RO_2_ conditions, the signals of the C_10_H_14_O_*z*_, C_10_H_16_O_*z*_, and C_10_H_18_O_*z*_ families were reduced by approximately 50%, 20%, and 40%, respectively (Figure 3). C_10_H_14_O_*z*_ monomers form exclusively via reactions of C_10_H_15_O_*x*_ with another RO_2_, so the sharp reduction in their relative abundance can be attributed to the diminished rate of RO_2_ cross-reactions (i.e., reaction 1). C_10_H_16_O_*z*_ monomers exhibited a less pronounced reduction because they formed via alcohol-producing RO_2_ cross-reactions ([Disp-formula eq1]) but also via RO_2_ + HO_2_ reactions that yield hydroperoxides ([Disp-formula eq4]) and should be enhanced under high HO_2_/RO_2_ conditions. C_10_H_18_O_*z*_ monomers may be either alcohols or hydroperoxides, depending on whether the termination reaction involves RO_2_ or HO_2_. C_10_H_18_O_7_ is the most heavily affected monomer – its signal strength increases by around 180% in the high HO_2_/RO_2_ regime (outlier in Figure 3). With seven O atoms, C_10_H_18_O_7_ can be considered a HOM. NO_3_^–^ CIMS gas-phase measurements showed that C_10_H_17_O_7_ was the dominant HOM-C_10_H_17_O_*x*_ RO_2_ in both HO_2_/RO_2_ regimes, but its signal was ≈10% stronger in the high HO_2_/RO_2_ case, as also found by Baker et al.^51^ This is likely due to increased hydroperoxide formation by the HO_2_ termination of C_10_H_17_O_7_. Another potential source of C_10_H_18_O_7_ is C_10_H_17_O_8_, which forms C_10_H_18_O_7_ alcohol upon reaction with RO_2_, though its contribution decreases under the high HO_2_/RO_2_ regime.

**Figure 3:**
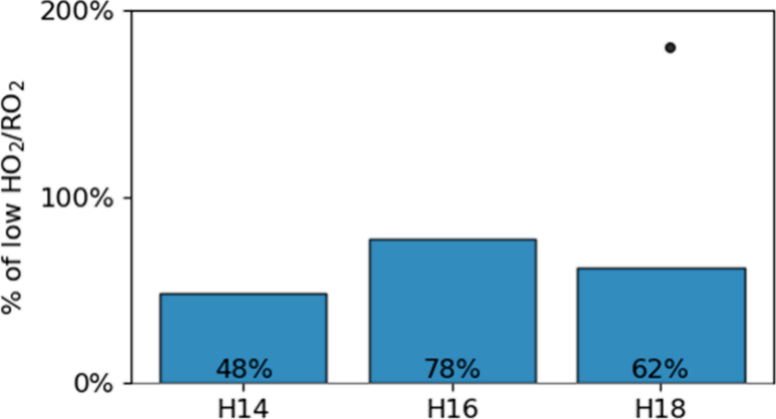
Differences in the particle-phase summed signal of α-pinene monomers from different hydrogen families (H_14,16,18_) between the high and low HO_2_/RO_2_ regimes. The outlier monomer in the H_18_ family is C_10_H_18_O_7_, which increases by 180% in the high HO_2_/RO_2_ regime. Data from exp. 1 and 2.

### 3.2 Effects on Oxidation Products Detected in Both Gas- and Particle-Phases

The effects of the high HO_2_/RO_2_ regime on particle-phase chemical composition measured by FIGAERO CIMS during seeded experiments (Exp. 1 and 2) was compared to the effect on gas-phase chemical composition detected by NO_3_^–^ CIMS during unseeded experiments. The NO_3_^–^ CIMS captures mainly HOM, which are typically considered condensable.^51,84,94,95^

In total, the FIGAERO CIMS detected 252 products in the particle phase, and the NO_3_^–^ CIMS detected 206 products in the gas-phase. Of these products, 89 were detected by both methods (Figure 4). The iodide CIMS detects molecules containing 2–10 oxygen atoms, while the nitrate CIMS extends this range to 6–18 oxygens, i.e., HOM, as previously reported by Riva et al.^96^ As a proportion of the overall molecular mass-weighted signal, the 89 products detected by both instruments comprised 37% of the NO_3_^–^ CIMS gas-phase and 38% of the FIGAERO CIMS particle-phase. The detection of some species exclusively by either I^–^ or NO_3_^–^ CIMS is attributed to differences in the clustering efficiency of the two reagent ions.

**Figure 4:**
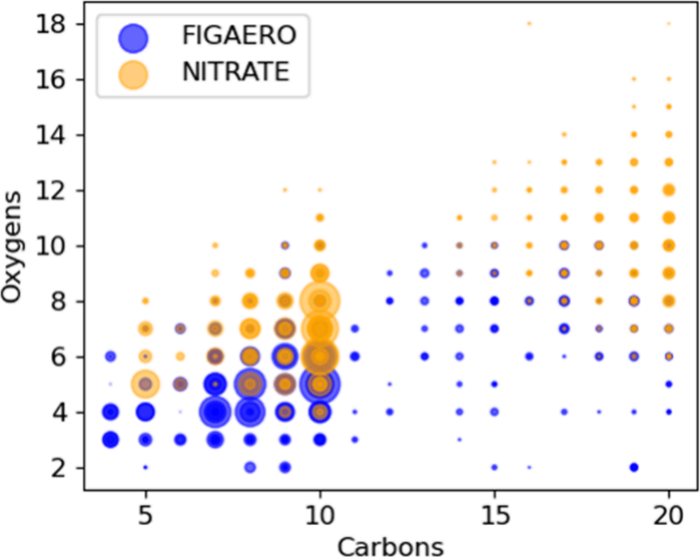
Distribution of α-pinene oxidation products under a low HO_2_/RO_2_ regime obtained with the FIGAERO I^–^ CIMS (particle-phase) and the NO_3_^–^ CIMS (gas-phase). Products are arranged by their numbers of carbons (*x*-axes) and oxygens (*y*-axes). The markers are sized based on the products’ signal intensity.

Figure 5 shows the effect for both gas- and particle-phases of the overlapping products to the change in the HO_2_/RO_2_ ratio. As noted in the Materials and Methods section, comparing the ratios of product signals between the two HO_2_/RO_2_ regimes effectively removes the influence of differences in the sensitivities of the two CIMS methods for individual products. Most of the overlapping products show similar changes in both gas- and particle-phase measurements, with deviations generally falling within a factor of 2 (Figure 5). The clustering of data near the 2:1 line indicates that the effect of the HO_2_/RO_2_ regime is reflected somewhat more strongly in the particle-phase. Some variability is expected since it is not obvious that the particle and gas-phases should respond identically upon changing the HO_2_/RO_2_ regime, e.g., for the particle phase, there is a potential for condensed phase reactions. Additionally, if several isomers are present (as is likely true), then the two detection techniques (I^–^ and NO_3_^–^ ionization) may respond differently.

**Figure 5:**
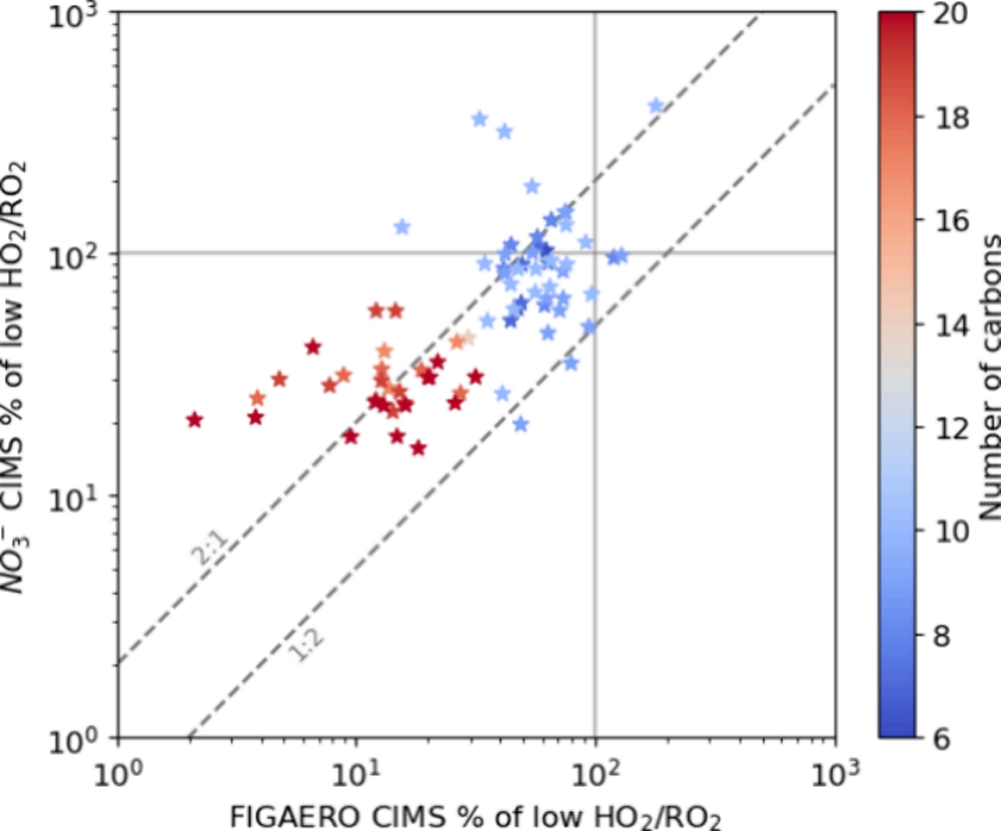
Effects of high HO_2_/RO_2_ conditions on oxidation products detected in both the gas- and particle-phases. On the *x*- and *y*-axes is shown the effect of the change in the HO_2_/RO_2_ regime for particle and gas phases, respectively, of the overlapping products (C6–10, 18–20). The effect is represented by the ratio of the products’ signal in the high HO_2_/RO_2_ to the low HO_2_/RO_2_ regime. The *x*- and *y*-axes are on a logarithmic scale. The *x* = 100% and *y* = 100% lines indicate the point where there is no change in the particle- and gas-phase signals, respectively. The color scale indicates the number of carbon atoms of the common detected products. FIGAERO CIMS data are from exp. 1 and 2. NO_3_^–^ CIMS data are from exp. 3 and 4.

Overall, the gas-phase measurements indicated that the high HO_2_/RO_2_ conditions reduced the monomers, fragments, and accretion products signals to 99 ± 5%, 94 ± 5%, and 32 ± 4%, respectively of the corresponding signal in the low HO_2_/RO_2_ regime. The given variability was calculated at one standard deviation. These values are comparable to those observed by Baker et al.^51^ in earlier gas-phase experiments (95 ± 4%, 81 ± 3%, and 41 ± 6%, respectively) using the same NO_3_^–^ CIMS.

### 3.3 Impact on SOA Formation Potential

Figure 6 shows the summed particle- and gas-phase molecular mass-weighted signal of the different product classes. Under both HO_2_/RO_2_ regimes, monomers and fragments provide the largest contributions to the particle-phase molecular mass-weighted signal, while accretion products provide the lowest contribution. The total particle-phase molecular mass-weighted signal under the high HO_2_/RO_2_ regime was 47% lower than that for the low HO_2_/RO_2_ regime (Figure 6A).

**Figure 6:**
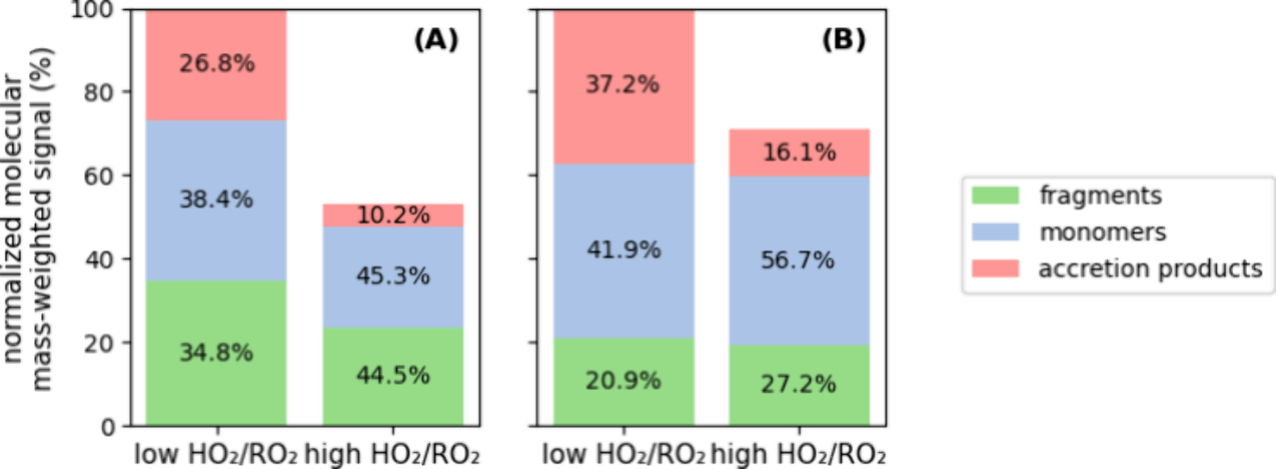
Molecular mass-weighted signals of α-pinene products in the particle and gas-phases under low and high HO_2_/RO_2_ regimes. The percentages indicate the contribution of fragments (green), monomers (blue), and accretion products (pink) to the total signal in each regime. The *y*-axes are scaled to the respective low HO_2_/RO_2_ summed molecular mass-weighted signal. (A) Particle-phase data from FIGAERO CIMS (exp. 1 and 2). (B) Gas-phase data from NO3- CIMS (exp. 3 and 4).

The measured total organic aerosol mass showed a 44% reduction from low to high HO_2_/RO_2_ regime, in line with the trend shown by the FIGAERO CIMS (47%) and the observation (27%) for similar experiments reported in Baker et al.^51^

The high HO_2_/RO_2_ regime reduced the monomer and fragment signal strength in absolute terms, as discussed above, but increased their relative contributions from 34.8% to 44.5% and 38.4% to 45.3%, respectively. Conversely, it reduced the relative contribution of accretion products from 26.8% to 10.2% while also reducing their absolute contribution.

Figure 6B shows the changes in the gas-phase species measured by the NO_3_^–^ CIMS, which are mainly HOM products. A 39% reduction is observed under the high HO_2_/RO_2_ regime compared to the low HO_2_/RO_2_ regime. HOM were expected to exhibit a lesser reduction than other conventional oxidation products because, as discussed above, autoxidation is faster than RO_2_ or HO_2_ terminations. Increasing the HO_2_/RO_2_ ratio raised the contribution of monomers from 41.9% to 56.7% and that of fragments from 20.9% to 27.2%, while that of accretion products decreased from 37.2% to 16.1%. Since HOM accretion products likely contain one or two HOM-RO_2_ entities, suppression of accretion product formation by HO_2_ should increase the abundance of monomers proportionately to the loss of accretion products. This was indeed seen for the hydroperoxide monomers in the gas-phase, but in the particle-phase, the shift from accretion products to monomers may not be reflected in the product distribution as the monomers can partition toward the gas-phase. While the accretion product distribution is reflected at about 1:1 in both phases, the increase of the monomers is smaller in the particle-phase. That could be the case if the monomers involved in dimer formation have lower vapor pressures than other monomers or are fragmented in the heating process. Interestingly, these changes do not significantly alter the fragment to monomer ratio, which is 1:1 under low HO_2_/RO_2_ conditions and 1.05:1 in the high RO_2_ regime.

### 3.4 Impact on Volatility

Based on the particle-phase measurements, the Tmax of individual compounds was used to derive their saturation concentration (log C*) by applying a relationship between Tmax and the parametrization of Peräkylä et al.^84^ (see Figure S4 in supplemental). Figure 7 (left panel) shows the resulting particle phase volatility distribution using the individual C* from the FIGAERO CIMS measurements. One may note that there are a number of other parametrizations presented in the literature. Applying any of these will shift the volatility distribution. For detailed discussion about differences between volatility parametrization, one may refer to the recent study by Ylisirniö et al.^89^

**Figure 7:**
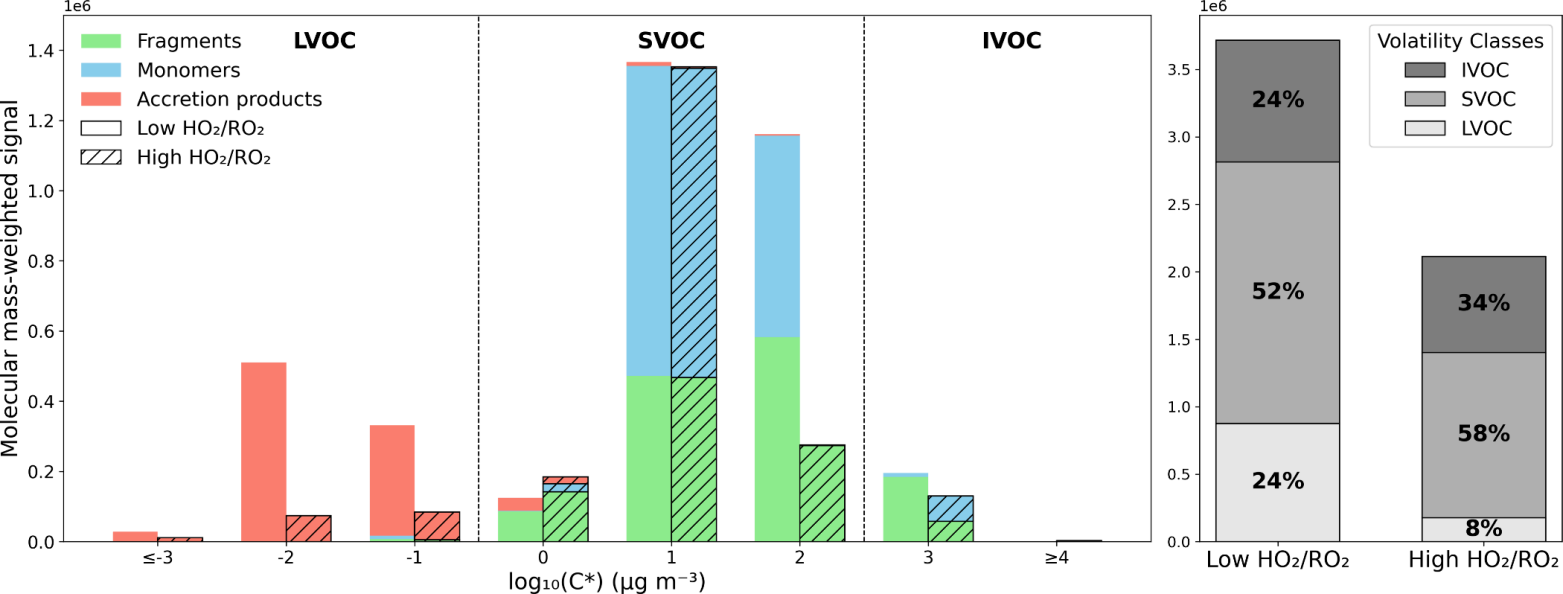
Particle-phase volatility distribution determined by FIGAERO CIMS. Right: molecular mass-weighted signal particle-phase product distribution, sorted into volatility bins using measured Tmax converted into log C* using Peräkylä et al.^84^ parametrization (see Figure S4 and associated discussion in supplemental). The bars represent the summed molecular mass-weighted signal of the products in each volatility bin under the low and high HO_2_/RO_2_ regimes (solid and diagonal patterns, respectively). The portions of the bars colored green, light blue, and pink indicate the contributions of fragments, monomers, and accretion products, respectively. The hatched bars represent the high HO_2_/RO_2_ regime, while the solid (nonpatterned) bars represent the low HO_2_/RO_2_regime. The log C* is divided into volatility regions:^70^ IVOCs for log_10_ C* > 2, SVOC for 0 < log_10_ C* < 2, and LVOC for log C* < 0. Left: Summed molecular mass-weighted signal of the products included in the volatility regions IVOC, SVOC, and LVOC from top to bottom under the low and high HO_2_/RO_2_ regimes. Data from exp. 1 and 2.

The detected product distribution in the particle-phase extends from saturation concentrations of 10^4^ to 10^–3^ μg m^–3^, hence, from IVOC (intermediate volatile organic compounds) to LVOC.^70^ The IVOC and SVOC regions are dominated by monomers and fragments, whereas the LVOC region is dominated by the accretion products. Although the dominant bin is log C* = 1 in both regimes, the volatility distribution in the high HO_2_/RO_2_ regime is weighed toward lower vapor pressures. While a significant fraction of monomers has log C* = 2 in low HO_2_/RO_2_, all the monomers are shifted to log C* = 1 in the high HO_2_/RO_2_. This volatility difference may arise from hydroperoxide monomers produced by RO_2_ + HO_2_ chemistry, which are generally less volatile than carbonyls and alcohols,^42,50,97^ which are produced by RO_2_ cross-reactions. The accretion products are the least volatile components and, as previously shown, are almost suppressed under the high HO_2_/RO_2_ regime. The volatility distribution in the high HO_2_/RO_2_ regime shows similar changes to those predicted by Schervish and Donahue^39^ in terms of reduced dimers in the saturation concentrations lower than 10^–1^ and increased ROOH levels.

Overall, the high HO_2_/RO_2_ regime yields products with a higher volatility than the low HO_2_/RO_2_ regime. The contribution of LVOC decreases from 24% to 8% from low to high HO_2_/RO_2_. On the other hand, the contribution from SVOC and IVOC increased from 52% to 58%, and from 24% to 34% (Figure 7, right panel). The bulk log C* values are rather similar for low and high HO_2_/RO_2_ regimes, −0.22 μg m^–3^ and −0.1 μg m^–3^, respectively. However, the volatility distribution shows that most of the signal shifts from LVOCs and IVOC to SVOC when enhancing the importance of HO_2_ chemistry.

This method of assigning products to volatility bins may be subject to bias because the volatility scale might shift between experiments if the total mass deposited on the filter differs varies widely.^98,99^ However, the total organic particulate mass produced under high and low HO_2_/RO_2_ conditions differs by only a few micrograms according to AMS measurements reported by Baker et al.^51^ Given the small mass accumulated on each filter, the differences between the experiments are considered negligible.

The observed reduction of the particle-phase under high HO_2_/RO_2_ concentrations is likely caused by a combination of both chemical and partitioning changes. Since the SVOCs contribute more substantially to the organic aerosol mass under a high HO_2_/RO_2_ regime (Figure 7, right panel), the total organic aerosol loading, which governs the gas-to-particle partitioning, becomes a more critical parameter. Baker et al.^51^ reported that the production of HOM decreased by 27% under a high HO_2_/RO_2_ regime. Because of their low volatility, HOM partition predominantly into the particle-phase when seed particles are present. In contrast, our study also includes SVOCs and IVOCs, whose partitioning is more sensitive to changes in the organic aerosol mass loading. To assess the latter, we estimated the effect of Raoult’s law on partitioning under the reduced SOA mass produced in the high HO_2_/RO_2_ regime. This analysis suggests that partitioning alone could explain a decrease of about 30% depending on the organic mass input. This indicates that partitioning effects may contribute significantly to the larger reduction observed with FIGAERO CIMS (47%) compared to the decrease in HOM reported by Baker et al.^51^ (27%), although the exact quantitative balance between chemistry and partitioning remains uncertain. The data presented in Figure 7 (left panel) illustrate how, despite the enhanced pathway toward monomers’ formation rather than accretion products, the mass of products in log C* = 2 and log C* = 3 is significantly lower under the high HO_2_/RO_2_ regime. This reduction can be partly attributed to the partitioning effect since the suppression of accretion products leaves less organic mass available for condensation under high HO_2_/RO_2_ conditions.

## Conclusions

Recent studies have highlighted the important role of the HO_2_/RO_2_ ratio in shaping the SOA composition. To address this, we designed a chamber study that compared SOA formation under an atmospherically representative HO_2_/RO_2_ regime (1:1) to that under a regime more commonly used in chamber studies (1:100). The oxidant level was maintained at the same level under both regimes. A FIGAERO I^–^ CIMS was used to characterize the particle-phase, while a NO_3_^–^ CIMS was used to measure gas-phase HOMs.

The enhanced importance of RO_2_ + HO_2_ reactions under the high HO_2_/RO_2_ regime strongly reduced SOA formation overall with a particularly pronounced reduction in accretion product formation. However, there was a very pronounced increase in the abundance of one hydroperoxide monomer (C_10_H_18_O_7_). Relative changes in the particle-phase product distribution observed in seeded experiments were compared with those seen in the gas-phase during unseeded experiments. Although two different ionization schemes were used for the particle-phase and gas-phase measurements, several ions with the same molecular composition were detected by both instruments, and they had a similar behavior when going from low to high HO_2_/RO regime. The molecular mass-weighted particle signal under the high HO_2_/RO_2_ regime was reduced by 47% when compared to the low HO_2_/RO_2_ regime, while the gas-phase signal was reduced by 39%. In both phases, these reductions were largely due to the reduced formation of accretion products. Previous studies have emphasized the importance of increased ROOH group formation based on the lesser volatility of hydroperoxides compared to other monomers. However, since the observed reduction of the particle-phase is likely due to a combination of both chemical changes and partitioning effect, the results obtained here suggest that increased hydroperoxide production did not offset the overall reduction in SOA under high HO_2_/RO_2_ conditions even though the majority of the monomers formed under such conditions had lower volatility than those produced in the low HO_2_/RO_2_ regime. Because accretion products, which are the least volatile components, are almost suppressed under the high HO_2_/RO_2_ regime, their absence is the primary reason for the higher volatility of SOA in this regime.

Thus, our study offers valuable insights into the influence of the HO_2_/RO_2_ ratio on α-pinene SOA formation under atmospherically relevant chamber conditions. It adds to the literature that clearly points toward a new methodology of doing chamber studies^51,52^ and the complexity in evaluating such systems,^35–38^ especially when it comes to investigating changes in radical chemistry.^39^ In addition to methodology and choice of conditions for experiments, the design using a stirred reactor was necessary to be able to scrutinize the particle phase composition and volatility distribution using the FIGAERO CIMS. To derive a more descriptive volatility distribution, our approach to apply the Tmax from our GUFIT procedure together with existing parametrized C* estimations showed great potential. Future research using similar approaches is necessary, particularly for mixed VOC systems, to better represent atmospheric complexity and to investigate how interactions among different RO_2_ radicals influence SOA formation, composition, and the volatility distribution of the resulting aerosol.

## Supplementary Material



## Data Availability

The data presented in this study are available from the researchdata.se database at the following link: 10.5878/kc6k-rh28.

## Notes

Additional details on FIGAERO data background subtraction; description of the GUFIT (Figures S1, S2, Table S1); scenarios of the impact of a high HO_2_/RO_2_ regime on EMG2, 3, and 4 (Figure S3); details on the volatility estimation (Figure S4) (PDF)

## 4

Leibniz-Institut für Troposphärenforschung e.V., Permoserstraße 15, 04318 Leipzig, Germany (Y.B.)

## 5

Thrust of Earth, Ocean and Atmospheric Sciences, Hong Kong University of Science and Technology (Guangzhou), Guangzhou 511453, China (Q.H.).

## 6

Swedish Meteorological and Hydrological Institute, 601 76 Norrköping, Sweden (M.P.).

## 7

Department of Earth and Environmental Sciences, University of Manchester, Oxford Road, Manchester M13 9PL, UK (R.W.).

## 8

Keplerstrasse 13, 35390 Giessen, Germany (T.F.M.).
