# Supplementary material for: Changes in Secondary Organic Aerosol Composition and Volatility Going from a Low to a High HO2/RO2 Regime in α‑Pinene Photooxidation
Source: ACS EST Air. 2025 Dec 3;3(1):104–15. doi: 10.1021/acsestair.5c00254 (PMC12797231; doi:10.1021/acsestair.5c00254)
Supplement: Supplementary file 1 [file ea5c00254_si_001.pdf]

Supplement of

Changes in Secondary Organic Aerosol Composition and Volatility Going from a Low to a High HO_2/RO_2 Regime in α-Pinene Photooxidation

Veronica Geretti[1], Yarê Baker[2,4], Thomas Bannan[3], Aristeidis Voliotis[3], Quanfu He[2,5],Thorsten Hohaus[2], Sungah Kang[2], Michael Priestley[1,6], Epameinondas Tsiligiannis[1], Hui Wang[2], Rongrong Wu[3,7], Annika Zanders[2], Sören R. Zorn[2], Gordon McFiggans[3], Cheng Wu[1], Thomas F. Mentel[2, 8] and Mattias Hallquist*[1]

Corresponding Author: Mattias Hallquist, hallq@chem.gu.se

[1]Department of Chemistry and Molecular Biology, University of Gothenburg, Gothenburg, 413 90, Sweden
[2]Institute for Climate and Energy Systems, ICE-3 Troposphere, Forschungszentrum Juelich GmbH, Juelich, 52428, Germany
[3]Department of Earth and Environmental Sciences, University of Manchester, Oxford Road, Manchester, M13 9PL, UK

S1 Background subtraction

Blank filter was sampled from the chamber when it was clean and free of reactants (containing only seed particles in the seeded experiments) for the same sampling time as sample filters. Because the particle-phase background signal is related to the signal intensity of the measurement, to correct for the particle-phase background, the blank filter was firstly scaled before subtraction: for each heating cycle, the average of its final thermogram data points was divided by the average of the corresponding points in the experimental filter. This ratio was used to scale the blank filter before it was then subtracted from the experimental filter.

S2 Gothenburg University FItting for Thermograms (GUFIT)

The FIGAERO multipeak thermograms were deconvoluted using the GUFIT (Gothenburg University FItting for Thermograms), a procedure written in Python (3.11.7) using Pandas (2.1.4), NumPy (1.26.4), SciPy (1.11.4), lmfit (1.3.2) libraries. The GUFIT utilizes Exponentially Modified Gaussian (EMG) functions to model the thermogram's shape (here, from 2 to 4)[1].

[4] Present address: Leibniz-Institut für Troposphärenforschung e.V., Permoserstraße 15, 04318 Leipzig, Germany
[5] Present address: Thrust of Earth, Ocean and Atmospheric Sciences, the Hong Kong University of Science and Technology (Guangzhou), Guangzhou, 511453, China
[6] Present address: Swedish Meteorological and Hydrological Institute, 601 76, Norrköping, Sweden
[7] Present address: Department of Earth and Environmental Sciences, University of Manchester, Oxford Road, Manchester, M13 9PL, UK
[8] Present address: Keplerstrasse 13, 35390 Giessen, Germany

$$\textbf{Eq. 1}\ \ f(x) = \frac{A}{2\tau}\, exp\left[\left(\frac{W}{2\tau}\right)^2 + \frac{T-x}{\tau}\right]\left[erf\left(\frac{x-T}{\sqrt{2}W} - \frac{W}{\sqrt{2}\tau}\right) + 1\right]$$

To find the multiple Tmax values in all thermograms, find_peaks function from scipy.signal was used with a prominence threshold of 1. This method was automated to scan through the signal intensity within predefined temperature ranges that roughly align with the observed peak locations. The other parameters' ranges were guessed. Eventually, the GUFIT utilizes the ranges to fit the model optimally.

After guessing a range for the A, T, W, and τ parameters for each EMG, the lmfit 1.3.2 package[2] with "powell" method, fits the multiple EMG functions to optimally describe the multipeak thermogram shape. The area of the fitted EMG functions is computed using the trapezoidal method and compared to the original data before applying the procedure. If the residual area exceeds 10% of the original data, the parameters must be modified and the fit repeated. The Tmaxes of the EMG1 areas are shown in Figure S1.

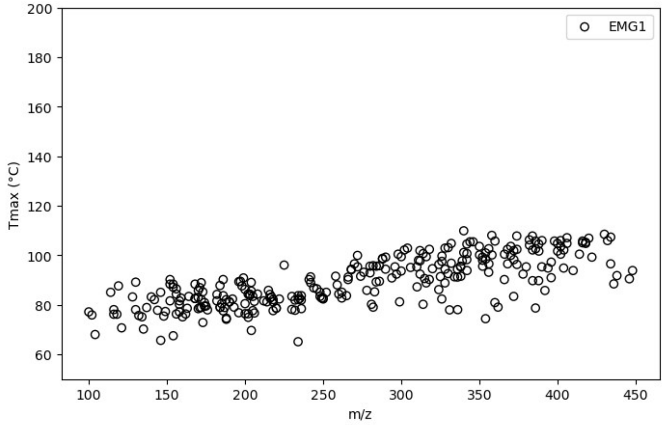

Figure S1: Tmax of EMG1 areas extracted from GUFIT application against the m/z of the detected compounds.

The Tmax ranges used in the GUFIT analysis were customized to the dataset. Specifically, different ranges were applied to fragments/monomers versus accretion products to better align with their respective desorption profiles. For accretion products with less multi-peak shape only 2 or 3 EMGs were applied (Table S1).

Table S1: Custom Tmax ranges (°C) used in GUFIT for monomers, fragments, and accretion products.

**Classes**	EMG1	EMG2	EMG3	EMG4
Fragments and monomers	60-90	91-110	110-135	136-200
Accretion products with 3 EMG	70-88	89-120	121-200	
Accretion products with 2 EMG	80-120	120-150		

In Figure S2, some examples of the GUFIT application are reported, one fragment, two monomers and one accretion product. It's important to note that the GUFIT model is a newly developed, custom-built Python code. It's well suited to extract data for EMG1 while the uncertainties increase for the following EMGs. Furthermore, the origin of these EMGs can have several causes. Therefore, this work focuses on data derived from EMG1 and only some initial characteristics for the remaining EMGs are described here in the supplemental.

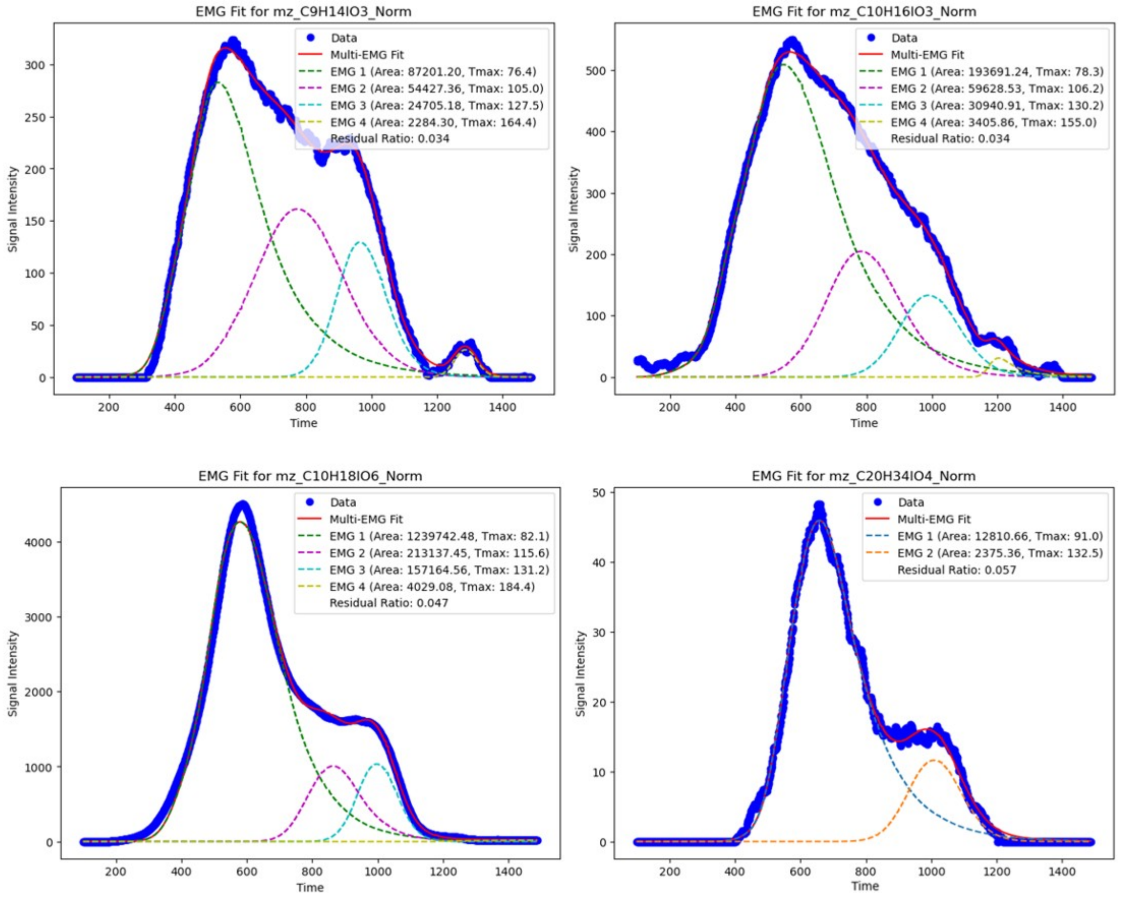

Figure S2: Examples of GUFIT deconvolution of different α-pinene oxidation products in the particle-phase.

S3 Scenarios of impact of high HO_2/RO_2 on SOA using GUFIT

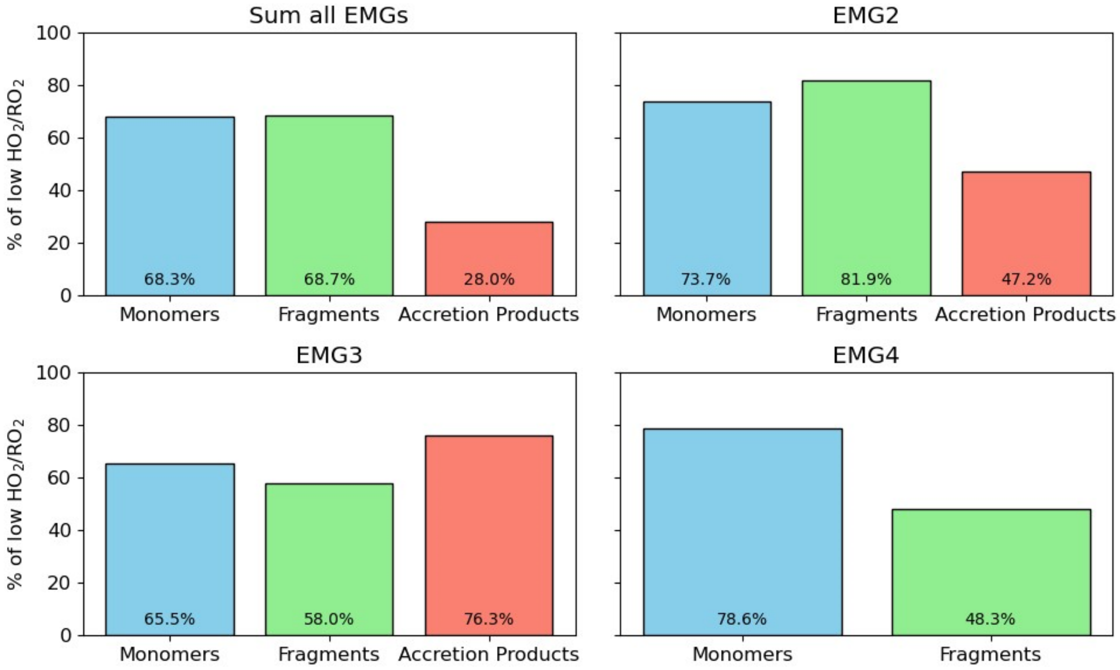

Figure S3: Scenarios of particle-phase summed signal using the EMG areas resulting from GUFIT application under the high HO_2/RO_2 regime relative to the low HO_2/RO_2 regime. The plots show monomers, fragments and accretion products. From top left to bottom right are shown the fractions of low HO_2/RO_2 regime of monomers, fragments, and accretion products using the different EMG areas. EMG1 is shown in the main manuscript.

The deconvolution of thermogram peaks using the GUFIT produced from 2 up to 4 EMG areas depending on the m/z investigated. The EMG1 area occupies, on average, 53% of the total thermogram area, the EMG2 area 23%, the EMG3 area 17% and the EMG4 area 7%.

The observation that secondary peaks can contribute ~50% of the desorbed area aligns with Lopez-Hilfiker et al. (2014)[3]. The impact of shifting HO_2/RO_2 regime on the different EMGs are shown in Figure S3. Compared to the EMG1, the summed EMGs areas and the EMG2&3 area show fairly similar conclusions for the monomers and fragments. The EMG4 area shows more reduction in the fragments compared to the others. The EMGs areas of the accretion products show more pronounced differences. Firstly, the EMG4 was not needed in the fitting of their desorption profile. Secondly, the reduction span from low to high HO_2/RO_2 regime was from 22% to 76.3%. Their EMG3 area shows the least reduction, however, it accounts for a small portion of the total particle-phase area.

The reason for complex desorption profile shapes can be due to different effects. The EMG2 peak is generally due to a broad description profile, which could be explained by the presence of isomers with different vapor pressures[4]. In our study, the Tmax of EMG3 peaks is very similar across the spectra, as similarly reported by Wang et al. (2018)[5], and a possible reason could be the thermal decomposition of thermally unstable oligomers[5–11]. EMG4, given its high Tmax is likely a peak associated with ammonium sulphate decomposition[5].

In summary, by restricting our evaluation and results to EMG1, we will ascertain that we do not incorrectly assign any changes in the particle-phase accretion products as fragments or monomers.

However, we acknowledge that we do not use the full capacity of the FIGAERO CIMS and will underestimate the amount of totally formed particle-phase products. Still, as all higher order EMGs show a decrease in their signals when going from low to high HO_2/RO_2 and likely consist of compounds with lower volatilities, it supports and strengthen our main conclusions on reduced low volatile material when enhancing the role of HO_2.

S4 Volatility estimation

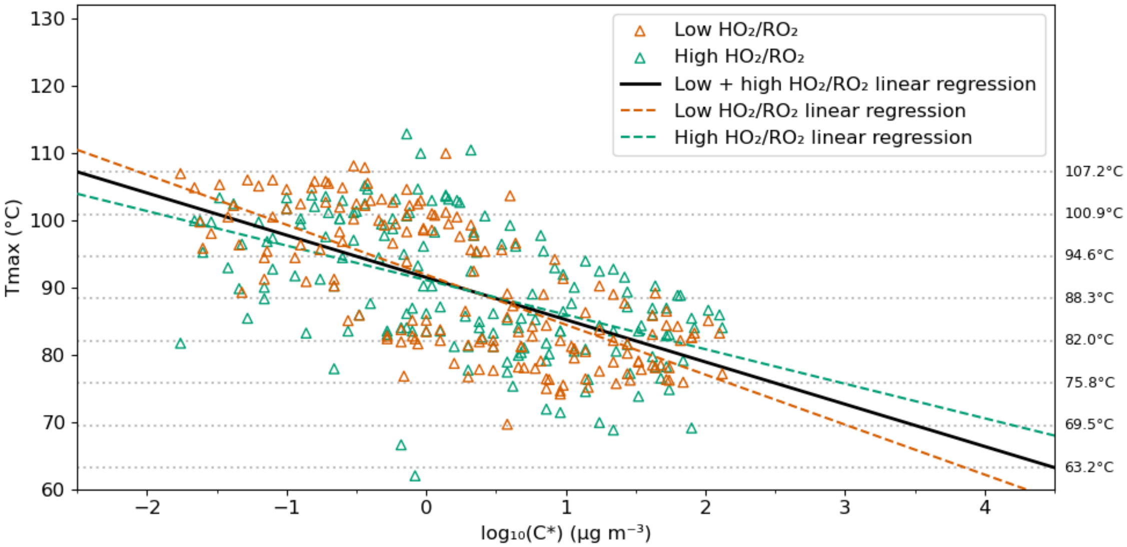

Figure S4: A-pinene OH oxidation products' Tmax from EMG1 in high and low HO_2/RO_2 (green and orange triangles, respectively), against the logC*[12]. The dashed green and orange lines are the linear regressions of the Tmax in high and low HO_2/RO_2 regime versus the logC*. The black line is the linear regression between all the Tmax versus the logC*.

Figure S4 shows the Tmax of the oxidation products products versus the C* as estimated using molecular composition and the parameterisation from Peräkylä et al. (2020)[12]. A liner regression was used to describe the relationship.

The data used excluded species that were assigned with a minimum double bond equivalent DBE > 3 (in total #6 species excluded representing less than 1% of the total ion count). The minimum DBE was calculated from the molecular formula as:

$$DBE = \frac{(2 \times nC + 2 - nH - 2 \times nO)}{2},$$

where nC, nH, and nO are the number of carbons, hydrogens, and oxygens of the molecule, respectively.

The relationship was similar for the two datasets, and the pooled (low HO_2/RO_2 + high HO_2/RO_2) relationship was used. From this relationship, individual C* can be calculated for each measured Tmax. This derived C* was used to bin the data and present a volatility distribution (Figure 7 in the main manuscript). Uncertainty of derived Tmax was estimated to be below 5°C, which is equivalent to about 1 order of magnitude of saturation concentration.

REFERENCES

1. Goodman, K. J. & Brenna, J. Thomas. Curve Fitting for Restoration of Accuracy for Overlapping Peaks in Gas Chromatography/Combustion Isotope Ratio Mass Spectrometry. *Anal Chem* **66**, 1294–1301 (1994).

2. Newville, M. *et al.* LMFIT: Non-Linear Least-Square Minimization and Curve-Fitting for Python. Preprint at https://doi.org/https://doi.org/10.5281/zenodo.12785036 (2024).

3. Lopez-Hilfiker, F. D. *et al.* A novel method for online analysis of gas and particle composition: Description and evaluation of a filter inlet for gases and AEROsols (FIGAERO). *Atmos Meas Tech* **7**, 983–1001 (2014).

4. Thompson, S. L. *et al.* Field intercomparison of the gas/particle partitioning of oxygenated organics during the Southern Oxidant and Aerosol Study (SOAS) in 2013. *Aerosol Science and Technology* **51**, 30–56 (2017).

5. Wang, D. S. & Hildebrandt Ruiz, L. Chlorine-initiated oxidation of alkanes under high-NO conditions: Insights into secondary organic aerosol composition and volatility using a FIGAERO-CIMS. *Atmos Chem Phys* **18**, 15535–15553 (2018).

6. D'Ambro, E. L. *et al.* Molecular composition and volatility of isoprene photochemical oxidation secondary organic aerosol under low- and high-NOx conditions. *Atmos Chem Phys* **17**, 159–174 (2017).

7. Lopez-Hilfiker, F. D. *et al.* Phase partitioning and volatility of secondary organic aerosol components formed from α-pinene ozonolysis and OH oxidation: The importance of accretion products and other low volatility compounds. *Atmos Chem Phys* **15**, 7765–7776 (2015).

8. Stark, H. *et al.* Impact of Thermal Decomposition on Thermal Desorption Instruments: Advantage of Thermogram Analysis for Quantifying Volatility Distributions of Organic Species. *Environ Sci Technol* **51**, 8491–8500 (2017).

9. Faxon, C., Hammes, J., Le Breton, M., Pathak, R. K. & Hallquist, M. Characterization of organic nitrate constituents of secondary organic aerosol (SOA) from nitrate-radical-initiated oxidation of limonene using high-resolution chemical ionization mass spectrometry. *Atmos Chem Phys* **18**, 5467–5481 (2018).

10. Schobesberger, S., D'Ambro, E. L., Lopez-Hilfiker, F. D., Mohr, C. & Thornton, J. A. A model framework to retrieve thermodynamic and kinetic properties of organic aerosol from composition-resolved thermal desorption measurements. *Atmos Chem Phys* **18**, 14757–14785 (2018).

11. Docherty, K. S., Wu, W., Lim, Y. Bin & Ziemann, P. J. Contributions of organic peroxides to secondary aerosol formed from reactions of monoterpenes with O 3. *Environ Sci Technol* **39**, 4049–4059 (2005).

12. Peräkylä, O. *et al.* Experimental investigation into the volatilities of highly oxygenated organic molecules (HOMs). *Atmos Chem Phys* **20**, 649–669 (2020).
